# Risk factors for anxiety and depression among pregnant women during the COVID-19 pandemic: A web-based cross-sectional survey

**DOI:** 10.1097/MD.0000000000021279

**Published:** 2020-07-24

**Authors:** Anna Kajdy, Stepan Feduniw, Urszula Ajdacka, Jan Modzelewski, Barbara Baranowska, Dorota Sys, Artur Pokropek, Paulina Pawlicka, Maria Kaźmierczak, Michał Rabijewski, Hanna Jasiak, Roksana Lewandowska, Dariusz Borowski, Sebastian Kwiatkowski, Liona C. Poon

**Affiliations:** a Department of Reproductive Health, Centre of Postgraduate Medical Education; b St. Sophia's Specialist Hospital; c Lazarski University, Faculty of Medicine; d Clinical Department of Obstetrics and Gynecology, Central Clinical Hospital of Ministry of Interior and Administration; e Department of Midwifery, Centre of Postgraduate Medical Education, Warsaw; f Institute of Philosophy and Sociology, Polish Academy of Sciences; g Department of Social Studies, Institute of Psychology, University of Gdansk; h Department of Family Studies and Quality of Life, Institute of Psychology, University of Gdansk; i Department Obstetrics and Gynecology, Pomeranian Medical University in Szczecin; j Clinic of Fetal-Maternal, Gynecology and Neonatology, Collegium Medicum, Nicolaus Copernicus University in Bydgoszcz, Poland; k Department of Obstetrics and Gynecology, The Chinese University of Hong Kong, Hong Kong, Hong Kong SAR.

**Keywords:** anxiety, coronavirus, COVID-19, depression, mental health, pandemic, pregnancy

## Abstract

**Introduction:**

The article presents a protocol of a cross-sectional study of mental health of pregnant women in relation to the coronavirus disease 19 (COVID-19) pandemic. The primary aim is to compare differences in anxiety and depression scores of pregnant women between countries affected by the COVID-19 pandemic. The secondary aim is to assess demographic, economic, and social aspects affecting maternal anxiety and depression scores among pregnant women worldwide in the time of the COVID-19 pandemic. Finally, we will be able to compare differences in perception of the different aspects of the COVID-19 pandemic (social distancing, restrictions related to delivery) between countries and according to the epidemic status (number of infected patients, number of reported deaths). The comparisons will also be done according to the COVID-19 status of the participants.

**Methods and analysis:**

It is a web-based anonymous survey of pregnant women living in countries affected by the COVID-19 pandemic. The survey is comprised of 3 sections:

1) Questions related to general demography, pregnancy health history, mental health history, socioeconomic factors, as well as perception of fear, burden and restrictions related to the COVID-19 pandemic;
2) General Anxiety Disorder-7 (GAD-7) questionnaire for anxiety assessment and;
3) Patient Health Questionnaire–9 (PHQ-9) for depression assessment.

Web-based recruitment for health research has proven to be cost-effective and efficient. At current times with the COVID-19 pandemic, limited resources and social distancing restrictions, performing a mental health study involving pregnant women on a large international scale cannot be safely conducted without involving social-media.

The fears of pregnant women fall into 3 categories: the medical condition, the economic status and the organization of daily activity.

The study has received approval of the medical ethics committee and has been registered on Clinicaltrials.gov. Results will be published in peer-reviewed journals and made public through all available media.

## Abstract 2

Supplemental Digital Content is available in the text

**Article Summary**

Strengths and limitations of this study

- The web-based design of the study will allow efficient, low cost data collection during the time of an international health care crisis.
- The survey is translated to more than 10 languages which will allow comparisons between many countries affected by the pandemic.
- Anonymous web-based surveys are prone to bias, but it is extensively discussed in the protocol how this bias will be managed during data analysis.
- The study protocol invites pregnant women to provide their personal information for participation in future research. This provides an opportunity for follow up of the participants.

## 1 Introduction

Anxiety is a feeling of worry, nervousness, or unease about something with an uncertain outcome and it can co-exist, predispose or cause depression.^[1]^ The unpredictability of the coronavirus disease 2019 (COVID-19) pandemic makes people prone to severe anxiety.^[2]^ Research has shown that pregnant women are especially prone to anxiety^[1,3]^ with prevalence of gestational anxiety between 15% and 23%,^[4]^ in comparison with 3 to 5% of anxiety symptoms in the general population.^[5]^

Risk factors for anxiety are similar in the general population and during pregnancy, including bad childhood experiences (overprotective or harsh parenting, maltreatment, and physical punishment). Parental history of mental disorders and low socioeconomic status have also been described to increase the risk of anxiety.^[2,5,6]^

About 10% of pregnant women experience perinatal depression globally.^[7–10]^ Untreated perinatal depression may result in adverse obstetric outcomes and is a risk factor for poor maternal health, inadequate prenatal care, and postnatal depression.^[10,11]^ In addition to the potential negative impact on pregnancy outcomes, perinatal depression is associated with disrupted maternal-infant bonding, increased irritability, and decreased activity. Children born to depressed mothers are at risk for delayed cognitive and language development, lower IQ, and increased prevalence of psychiatric and emotional problems. Depression that begins during pregnancy frequently continues or worsens after delivery.^[7,9,12]^

Mothers have a strong desire to provide their children with a stable environment, however, the situation of the growing pandemic with strict limitations regarding social contacts and economic instability does not make a secure situation for procreation.^[5]^ Based on the current data, it seems that the severe acute respiratory syndrome coronavirus 2 (SARS-CoV-2) does not cause a severe course of infection in pregnant women as they are usually young and without co-morbidities.^[6]^ It is worth to underline that the COVID-19 pandemic is not only a public health crisis but also a social, demographic and economic one and it has a substantial negative psychosocial effect on everyone, including pregnant women. The resulting anxiety of pregnant women has a negative impact on pregnancy, such as increased risk of preeclampsia, depression, nausea, vomiting and could even cause preterm labor or miscarriage.^[1]^ What is more, maternal anxiety may lead to adverse effects of the newborns, like low birth weight, growth restriction or low APGAR score.^[13,14]^

The primary aim of our study is to compare differences in anxiety and depression scores of pregnant women between countries affected by the COVID-19 pandemic. The secondary aim is to assess demographic, economic, and social aspects affecting maternal anxiety and depression scores among pregnant women worldwide in the time of the COVID-19 pandemic. Finally, we will be able to compare differences in perception of the different aspects of the pandemic (social distancing, restrictions related to delivery) between countries and according to the epidemic status (number of infected patients, number of reported deaths). The comparisons will also be done according to the COVID-19 status of the participants.

## 2 Methods and analysis

### 2.1 Study design

This is a cross-sectional study. Data will be collected through an anonymous web-based survey made up of closed questions with multiple choice answers. The survey has three parts:

1) questions related to general demography, pregnancy health history, mental health history, socioeconomic factors, as well as perception of fear, burden and restrictions related to the COVID-19 pandemic and
2) General Anxiety Disorder-7 (GAD-7) questionnaire for anxiety assessment
3) Patient Health Questionnaire–9 (PHQ-9) for depression assessment.

#### 2.1.1 Survey development

The survey will be conducted using the Research and Electronic Data Capture (REDCap) tool. It is a secure application that provides an interface for data entry.^[15,16]^ All data will be collected anonymously, and the answers will not be attributed to a specific individual.

The survey has been developed by a multidisciplinary team comprised of physicians, midwives, psychologists, and sociologists. All experts have proposed questions from their field. The questions, in turn, have been reviewed by a second group of field experts. The second group of experts also proposed questions, and their feedback has been included in the final version of the survey. A pilot study was performed on ten Polish, Chinese and English-speaking pregnant women, and their feedback was obtained to enhance the readability and the content of the survey. In the following stage of the study, this will be done for all participating languages.

#### 2.1.2 Description of the survey

The survey collects information on demography, socioeconomic situation, general health history, pregnancy risk assessment, mental health history, and specific aspects related to the COVID-19 pandemic. Both GAD-7 and PHQ-9 scales are used to assess anxiety and depression. The survey consists of 60 questions, and it has the following structure: screening questions, consent form, demographic and socioeconomic questions, mental health history questions, general health history questions, pregnancy risk assessment questions, COVID-19 specific questions, and the GAD-7 and PHQ-9 scales. Supplemental Digital Content (Appendix 1. Full survey in English)

#### 2.1.3 Screening questions

The survey has 6 screening questions, of which the first asks if the woman is pregnant or not. It is followed by questions regarding last menstrual period and estimated date of delivery, type of provided care (midwife, doctor, shared, none), and if the pregnancy has been confirmed by a medical professional. This is aimed at decreasing bias created by women claiming to be pregnant to complete the survey.

Demographic and socioeconomic questions ask about place of residence, education, number of people in household, sources of income, marital status, and family/partner support. Pregnancy risk assessment questions include parity, previous mode of delivery, pre-pregnancy chronic diseases, pregnancy-related complications, and infertility treatment. Mental health history questions cover pre-pregnancy and current mental health issues. COVID-19 specific questions cover fear and burden related to COVID-19 pandemic and the restrictions imposed in order to limit the spread of the virus.

#### 2.1.4 Consent form

Once participants are deemed eligible to participate, a consent form appears that explains the purpose of the study, what the data will be used for, how the data will be stored, requirements of the participant, and any potential benefits or risks incurred by participating, and that consent for participation can be withdrawn at any point before submission of the questionnaire. Because of the questionnaire's anonymous nature, the participant information cannot be withdrawn after data has been submitted. The researchers will not know which data belong to which participant. Participants will be informed of this in the consent form.

#### 2.1.5 GAD-7 scale

Generalized Anxiety Disorder 7-item (GAD-7) questionnaire measures the severity of anxiety over the past two weeks. The GAD-7 questionnaire is a 7-question scale. There are four choices regarding the severity of symptoms: “not at all”, “several days”, “more than half the days” and “nearly every day”, which correspond to 0, 1, 2, and 3 points score, respectively (minimum total score 0, maximum 21).^[17]^ The summarized score is used to assess anxiety intensification. The scale is commonly used to diagnose generalized anxiety disorder in the general population. National Institute for Health and Care Excellence (NICE) recommends using this tool as a measurement of prenatal anxiety.^[18]^ The GAD-7 scale is the only scale besides the State-Trait Anxiety Inventory (STAI) that is validated for women in the prenatal period.^[4]^ The advantages of the GAD-7 scale are that it is more straightforward, easy to complete and that the interpretation is easier with fewer questions: 7 in GAD-7 vs. 20 in STAI. Moreover, the GAD-7 scale is available without cost and is easier to use on social media. In the general population a score of ≥ 10 allows diagnosis of generalized anxiety disorder.^[19]^ Nevertheless, in pregnant women, the cut-off point has been established as ≥7 with a sensitivity of 73.3% and specificity of 67.3%.^[20]^ (Table 1)

**Table 1:**
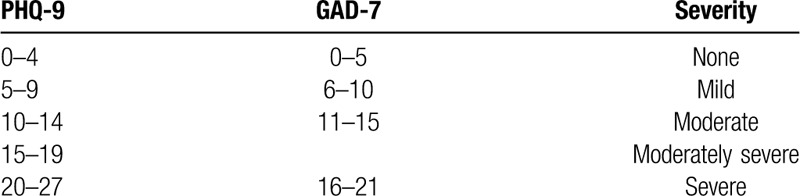
GAD-7 and PHQ-9 scale – anxiety and depression.

#### 2.1.6 PHQ-9 scale

NICE also recommends using the PHQ-9 scale with the GAD-7 scale to measure the depression risk in pregnant women.^[18]^ PHQ-9 has a similar grading system to GAD-7 and consists of nine questions exploring the depression symptoms over the past two weeks with four possible answers: “not at all,” “several days,” “more than half the days” and “nearly every day,” which correspond to 0, 1, 2, and 3 points score, respectively (minimum total score 0, maximum 27).^[21]^ According to American College of Obstetricians and Gynecologists (ACOG), Edinburg Postnatal Depression Scale (EPDS) and PHQ-9 are appropriate tools to measure antepartum depression.^[22,23]^ The advantage of the PHQ-9 scale is that it has fewer questions: 9 questions vs 20 in EPDS. Participants that achieve a score of ≥ 10 meet diagnostic criteria of perinatal depression.^[24]^ (Table 1)

#### 2.1.7 Follow-up, disclaimers and data storage

After completing the questionnaire, women will be given the option of providing their contact information (first name and email address). This database of contacts will be used to update participants on the results of this study and invite them to future studies and activities. Neither the women who participate in giving their data nor those that provide contact information in the study will receive any compensation. This study has received no funding and is a non-profit initiative of an international perinatal medicine community to better understand the burden of COVID-19 pandemic on maternal mental health worldwide. Contact information will be collected using a separate form so that personal information cannot be linked to the data provided in the web-based survey.

In the consent section of the survey, participants will be warned that some questions might be of a sensitive nature, and they can skip any question that they do not feel comfortable answering. The only parts of the survey that need to be fully answered are the GAD-7 and PHQ-9 scale.

All data will be stored on secure internal servers at the St. Sophie's Obstetrics and Gynecology Hospital, Warsaw, Poland, and will be directly downloaded from the REDCap tool and stored on a password-protected computer in a locked room at the Department of Reproductive Health of the Centre of Postgraduate Medical Education located on the hospital grounds. Data will be stored in this location for 3 years before it is destroyed. Principles outlined in the Data Protection Act are considered to ensure proper handling of the collected personal data.

#### 2.1.8 Recruitment

Pregnant women will be recruited through social media to complete a web-based survey. Inclusion criteria are the following: declaration of being pregnant, being able to complete the survey in the available languages (English, French, Spanish, Chinese, Polish, German, Russian, Italian, Ukrainian, Czech, Swedish, Albanian, Hebrew, Arabic, and Norwegian), answer the screening questions and provide informed consent for participation.

Exclusion criteria are the following: not providing online informed consent for participation or if the participant does not click on the submit button at the end of the survey, and women who do not answer all the GAD-7 and PHQ-9 scale questions.

#### 2.1.9 Sample size

The estimated sample size is 500 for each country. This has been calculated using the CinCalc.com sample size calculator.^[25]^ Results of GAD-7 for the general population vary between 4,75 (+/−4.76) to 5,7 (+/− 4.9).^[20,26]^ For PHQ-9, the scores vary from 3,0 (+/−4,3; for population in age of 30–39) to 5,2 (+/− 5.06).^[26,27]^ To detect a 20% difference separately in anxiety (GAD-7 scale) and depression levels (PHQ-9 scale) between the studied countries using a power of 80% and a margin error of 0.05,^[25]^ the calculated sample size for GAD-7 is 394 in every single country and for PHQ-9 calculated sample size is 372 in every single country. Values yielding higher sample sizes will be used. This sample size will allow us to compare reported anxiety and depression levels among pregnant women in different countries around the world affected by the pandemic.

#### 2.1.10 Data collection

The Pregmind International Research Group was formed, and a website was constructed for the international promotion of the study (www.pregmind.org).^[28]^ Data collection will begin with an initial social media post created on the the Pregmind Facebook fan page. The post will be prepared in the predefined languages and shared publicly. Campaigns will be set up to promote the study. Advertisements and posts will be shared on pages targeted towards pregnant women on every continent in the world in the predefined languages. We will also target closed pregnancy orientated groups via private messages on Facebook and invite them to post the study advertisement with the link to the survey on their pages.

The recruitment target will be reached when the desired sample size per region is achieved. We hope to collect data from at least one country from every continent. The survey will be translated into 15 languages, out of which 4 (English, Spanish, Chinese, Arabic) are widely spoken around the world and are not nationality related. For this study, the recruitment rate is difficult to predict. We expect data collection to take place at least six months for each language, based on similar previous studies of mental health.^[29]^ Recruitment rates may vary in different regions. Recruitment will not start simultaneously for all regions.

#### 2.1.11 Statistical analysis

Data will be kept anonymous and non-identifiable. For the statistical, analysis, R software^[30]^ and the Mplus computer program will be used.^[31]^ Data collection will include the most relevant demographic variables allowing us to compare collected data within a population. Reweighting and other techniques will allow adjustment for known or expected discrepancies between sample populations.^[32]^ To ensure comparability reweighting techniques, measurement invariance analysis will be performed. For the reweighting, we will use procedures suggested by Gelman and Carlin.^[32]^ We will collect external information about the distribution of age, place of residence, education level of pregnant women in the population, and adjust our sample to match the proportion in each country. If this is not possible because of the shortage of data, we will use propensity score matching^[33]^ and regression technique to balance the groups’ demographic distributions and make comparisons justifiable. Additionally, to ensure a valid comparison between countries, we will conduct a detailed measurement invariance analysis.^[34]^ Furthermore, we plan to perform regression analyses to explore potential associations between the reported anxiety, depression levels, and medical, sociodemographic, and pandemic related variables (including burdens related to social distancing). Altogether there will be 30 different variables. According to a rule of thumb “N > 104+m”, where N is the number of participants and m is the number of variables, the sample size will allow us to perform all planned analysis.^[35]^ Our main variables (GAD-7 and PHQ-9 scale) are measured by multiple indicators (questions), this opens the possibility to use Confirmatory Factor Analysis (CFA). This technique will allow us to assess the comparability of variables and, if necessary, address the problem of comparability using different latent variable modeling strategies.^[34]^ Principal analysis will be conducted using Structural Equation Modeling Framework which allows us to account not only for comparability problems but as well for measurement error.^[36]^

#### 2.1.12 Ethics and dissemination

The study protocol is in accordance with and was based on the Helsinki Declaration. Ethics approval was obtained from the Medical Bioethics Committee of the Centre of Postgraduate Medical University (Decision No. 56/PB/2020). The study has been registered at Clinicaltrials.gov, registration number NCT04377412. Participation in the study is voluntary and anonymous. Participants consent to the study by filling a web-based survey and clicking the submit button at the end. Before beginning they are informed that they can withdraw at any time and none of the information will be stored in the database. Participants may provide their personal information for participation in future research, but this information will not be linked with the data provided in this study.

The results of the study will be presented at local, national and international conferences related to mental health, pregnancy and COVID-19. Data will be published in peer-reviewed journals. The main findings of the study will also be shared and disseminated to researchers, health service providers, healthcare professionals and the public, especially pregnant women through all forms of media and communication.

## 3 Results

Web-based recruitment is not free of problems, but it is time-efficient and allows for fast data collection in cross-national settings. Typical survey procedures require months of preparation, and realization is not free of obstacles. Despite the potential problems, web-based surveys have proven to be useful in many types of projects,^[37]^ including assessment of mental health as well as other aspects of pregnancy, for example, nutritional habits.^[38]^ In this study data collection procedures are designed to maintain the highest degree of cross-country comparability.

Web-based recruiting for health research has proven to be cost-effective and efficient.^[39]^ At current times with the COVID-19 pandemic, because of limited resources, and social distancing restrictions, performing a mental health study involving pregnant women on a large international scale cannot be safely conducted without involving the social-media.

A web-based survey can be prone to several types of bias, such as non-response bias, response bias, selection bias, recall bias, and volunteer bias.^[40]^ Although the medium used to propagate the survey is available both in developed and underdeveloped countries, there is a risk that our survey will reach more women of a higher socioeconomic status and from larger agglomerations. To some extent, we could control this bias using historical data collected by conventional methods. In our study, additionally, bias will be accounted for by planning the campaigns for the dissemination of the survey to reach populations in various settings. Response-bias, on the other hand, is a situation when there is a systematic distortion in the way respondents answer the questions. This kind of bias is mainly a problem in surveys that investigate socially unacceptable/embarrassing behavior, that is, alcohol consumption or illegal drug use. Although this is not the case, there is a risk that pregnant women are particularly worried about the COVID-19 pandemic and will be more likely to respond to the advertisement of a survey assessing mental health related to the COVID-19 pandemic. This type of bias is also defined as recall bias, which is often the case in medical surveys where respondents who are prone to a specific sickness, are sick, or are more interested in the disease, are more likely to respond to a survey.^[41]^ For this reason, we will collect background information regarding mental health problems and previous treatments to distinguish such responses. We expect a higher incidence in difficulties with coping with the COVID-19 pandemic in women with a previously diagnosed mental health problem. It has been described that the experience of having the studied condition encourages an ’effort after meaning.’ The participant in such a case has already gone over his/her life history to understand why he/she has become ill. This clearly predisposes to recall bias.^[41]^ Again, this is not the case in our study. Volunteer bias would have occurred if there is a systematic difference between those who volunteer to be part of the study (completed the survey) and the population. In this case, we use a convenience sampling method, which involves sampling participants who are available to be approached at the time of recruitment. Also, we are approaching a specific part of the population – pregnant women. If there were a significant difference between those patients selected for the survey and those who are not, this would result in a sample that is not representative of the population. Convenience sampling is a proven, efficient, cost-effective method of recruitment for a web-based survey.^[42]^ The study aims to assess differences between countries resulting from the COVID19 pandemic. It will be hard to assess if the presented views will reflect the views of the general population of pregnant women in specific countries. This can be accounted for by analyzing the spread of the demographic and socioeconomic factors in the studied populations. The strength of the study is that we are targeting unselected pregnant women.

## 4 Discussion

### 4.1 Mental health impact

As a result of COVID-19 related mortality and morbidity, the rapid spread of the virus worldwide, WHO (World Health Organization) announced a pandemic state on 12 March 2020. As a result of the announcement, but also prior to the announcement, governments worldwide implemented measures to avoid further spread of the virus and reduce the number of causalities.^[43]^ Because of the pandemic, many essential services that usually make life easier, like banking, shopping facilities, all kinds of services, including medical, childcare, schools, sports and entertainment are unavailable due to new regulations. Many people are asking themselves questions such as: “How long will all this last?”, ‘How will it impact my life in the long-term?”, “Will I have a job when this is over?”, “Will I have an employer to return to?”, “Will all my family members survive?” and many more. Isolation and social distancing are important risk factors of mental health.^[44–46]^

### 4.2 Pregnant women and their unborn babies

Fetal well-being is one of the main maternal concerns. Although the possibility of vertical transmission has not been confirmed with concrete evidence, women may feel worried about such risk or infection of the infant in the peripartum period. Pregnancy is also the time of increased medical observation, which is difficult to facilitate during the pandemic.^[47]^ The difficulty in accessing professional medical help may also be a source of anxiety for pregnant women.^[48]^ Besides, pregnant women may feel insecure about exposure risk to the coronavirus when accessing medical facilities.

### 4.3 Existing children and family members

Since schools have been closed, the 24-hour presence of children at home is an additional source of stress because of the additional time dedicated to looking after them, the lack of physical/outdoor activities and the need to provide home-schooling. Financial problems and newfound duties related to family care can potentially lead to misunderstandings between family members. The support of the partner has a significant influence on maternal well-being and therefore single mothers may be more prone to anxiety.^[49]^

### 4.4 Societal impact

The COVID 19 pandemic has resulted in increased fear and uncertainty. This in turn may lead to negative societal behaviors. This may include behaviors aimed at regaining control of the situation, such as clearing shelves at supermarkets resulting in temporary shortages of food or other essentials such as toilet paper.^[50]^ More and more countries are implementing mass quarantines. The experience of isolation, fear of being trapped and rumors spread on social media can all result in growing anxiety and social panic.^[50]^ Other aspects of social life are being severely affected as well, such as family gatherings, participation in holidays, religious celebrations, births, funerals and many more. All of these can lead to growing anxiety and depression.^[51]^ Another important social aspect is the fear of blame, guilt and stigmatization related to being infected with COVID-19. Infected people may become a target of discrimination. There have been documented incidents of suicide due to guilt related to being infected as well as to social media bullying of medical professionals.^[52,53]^

### 4.5 Economic impact

The COVID-19 pandemic has affected the job market. Salaries have often been reduced and many people have lost their jobs leading to difficulties in paying bills and loans, and problems with daily survival costs. Measures to contain the virus directly influence economy. In China where the outbreak struck first, production fell by 13.5% and some sectors have collapsed completely, such as car sales by 92% and restaurant sales by 95%. The predicted decrease of the US economy in the second quarter of 2020 is 24% as a result of the COVID-19 pandemic.^[54]^ More than a decade ago the world suffered an economic crisis similar to the one we are expecting to happen now. That global financial crisis resulted in declining mental health worldwide with a rising number of suicides, alcohol and drug related deaths.^[54]^ The intricate vine of dependencies between the safety of all people, economy, health and political systems shows how vulnerable we have all become during these difficult times.

## 5 Conclusion

The COVID-19 pandemic has shown us that in an extreme environment mental health is a major public issue. The crisis that we are facing now, if appropriately managed, may become an opportunity for a global social transformation of perceptions, and interventions are needed to address mental health.^[55]^ We hope that our study, with its international reach, issues to be addressed and data to be gathered, will play a role in this process.

## Acknowledgments

We would like to thank Żelazna Medical Centre for their support in this project.

## Author contributions

AK – conceived the study, designed the study protocol, drafted the manuscript; SF - drafted the manuscript; UA - designed the study protocol, drafted the manuscript; JM - designed the study protocol, drafted the manuscript; BB - conceived the study, designed the study protocol,

DS - conceived the study, designed the study protocol, drafted the manuscript, organized and implemented the REDCap program; AP – designed, revised the study protocol, planned and wrote the statistical analysis of the study; MR – participated in drafting and revising the manuscript; HJ - designed the study protocol; RL - designed the study protocol; DB - conceived the study, designed the study protocol, participated in the local networking of the study

SK - conceived the study, designed the study protocol, drafted the manuscript, participated in the international networking of the study; LP - drafted and critically revised the study protocol and manuscript, participated in the international networking of the study

## Supplementary Material

Supplemental Digital Content
