# Supplementary material for: Risk factors for anxiety and depression among pregnant women during the COVID-19 pandemic: A web-based cross-sectional survey
Source: Medicine (Baltimore). 2020 Jul 24;99(30):e21279. doi: 10.1097/MD.0000000000021279 (PMC7387043; doi:10.1097/MD.0000000000021279)
Supplement: Supplemental Digital Content [file medi-99-e21279-s001.docx]

**Survey Introduction:**

The survey takes about 10-15 minutes to complete. Your participation in the survey is absolutely voluntary. All the answers you give will be kept entirely confidential as the survey is anonymous and we will not be able to identify you.

If you have any questions please feel free to contact our research team at the following e-mail address: [mamaCOVID19@prenatalprojekt.pl](mailto:mamaCOVID19@prenatalprojekt.pl)

We will begin with a few short questions to determine if you are eligible to participate.

**Screening questions:**

1. Are you pregnant?
   1. Yes
   2. No (if NO, cannot continue)
2. Please enter your birth of date: <date from calendar>
3. Please enter the date of your last menstrual period: <date from calendar> <option: date not available>
4. Please enter your due date: date from calendar
5. Have you confirmed your present pregnancy at a medical facility?
   1. Yes
   2. No
6. Who is managing your prenatal visits?
   1. Doctor
   2. Midwife
   3. Shared doctor and midwife
   4. Neither

**Survey ineligibility:**

Thank you very much for your interest in our survey. You are not eligible for this study at this time. Please feel free to contact our research team if you have any questions:

[mamaCOVID19@prenatalprojekt.pl](mailto:mamaCOVID19@prenatalprojekt.pl)

Thank you for your time.

You can leave the survey by exiting the page.

**Survey consent form:**

Thank you, you are eligible to participate! Before you start answering the questions please read the following consent form.

The following form explains the study in which you are being asked to participate. Please read it carefully and contact the investigators below if you have any questions before you provide your consent to participate. Contact us via e-mail at: [mamaCOVID19@prenatalprojekt.pl](mailto:mamaCOVID19@prenatalprojekt.pl)

Title of the Research Project:

**Risk factors for anxiety and depression among pregnant women during the COVID-19 Pandemic**

**Principal Investigator:**

**Anna Kajdy MD, PhD**

**Co-investigators:**

**Stepan Feduniw, Jan Modzelewski, Barabara Baranowska, Dorota Sys, Artur Pokropek, Sebastian Kwiatkowski, Urszula Ajdacka, Liona C. Poon**

Your participation in this survey is absolutely voluntary. You have the right to withdraw your participation at any time before clicking the SUBMIT button at the end of the survey. To withdraw simply exit the page. None of the answers will be collected unless you click the SUBMIT button. This survey is anonymous. Anonymous means that once you submit your answers the research team will not be able to identify which data is yours and your data cannot be withdrawn.

By clicking the SUBMIT button at the end of the survey you are giving your consent to participate in the study. You are not waving any of your legal rights as a research participant by consenting to participate in the study.

In this study, you are being asked to complete an online survey as accurately as possible. The survey takes approximately 10-15 minutes to complete. The survey includes questions about demography, socioeconomic situation, general health history questions, pregnancy risk assessment, mental health history and specific aspects related to the COVID19 pandemic. It also includes two standardized scales GAD-7 and PhQ-9 to assess your symptoms of anxiety and depression.

You may also choose to provide us with your name and contact information at the end of this survey if you are interested in participating in future studies. This information will be collected on a separate page from the survey and will not be linked to the information you have provided in the survey.

Benefits:

This study is being conducted by an international team of researchers. The aim is to better understand the aspects of pregnant women’s mental health during the COVID19 pandemic. We have not received any funding and therefore are not able to compensate you in any form. We hope that your participation and the information provided will help us plan interventions for pregnant women during this worldwide public health crisis.

Risks:

This survey is anonymous, and the information provided cannot be linked back to you. Your Internet Protocol (IP) address will be collected by the survey tool but the research team will not have access to this information. There is a small risk of third party’s interference. A result of such interference the information that you provide in the survey could be traced back to you through your IP address. To minimise this risk this survey is hosted on a secure server at the St. Sophie’s Obstetrics and Gynaecology Hospital secure server. It is the same server that contains all the hospital’s patient information, so it is very secure.

The questions in this survey among others ask about your current and prior mental health issues (if any) and your socioeconomic situation. Some of the questions may make you feel uncomfortable, upset and you may not want to answer them. You may skip those question(s) you do not want to answer or exit the survey and therefore withdraw your consent for participation in the study.

Data storage and protection:

Your privacy and confidentiality are very important to us and they will be protected throughout the study. All electronic data (the answers that you provide to the questions in the survey) will be stored on a password-protected computer in a locked a room at the Department of Reproductive Health of the Centre of Postgraduate Medical Education located on the grounds of the St. Sophie’s Specialist Obstetrics and Gynecological Hospital in Warsaw, Poland. Data will be stored in this location for 3 years before it is destroyed.

If you have any questions about our research please contact the research team at: [mamaCOVID19@prenatalprojekt.pl](mailto:mamaCOVID19@prenatalprojekt.pl)

This survey study has been approved by the Centre of Postgraduate Medical Education Research Ethics Committee (Project No. 56/PB/2020).

**Survey Questions:**

**DEMOGRAPHIC AND SOCIOECONOMIC QUESTIONS:**

1. Country/Region/City of residence: Pick from a list of countries/regions/cities, include all world countries
2. Which of the following best describes where you live at this time?
   1. A rural area (population of less than a 1000)
   2. A small population centre (population between 1000-29 999)
   3. A medium population centre (population between 30 000 – 99 999)
   4. A large population centre with (population between100 000 – 499 999)
   5. A very large population with population over 500 000
3. Which of the descriptions below comes closest to how you feel about your household’s income nowadays?
   1. Living comfortably on present income
   2. Coping on present income
   3. Finding it difficult on present income
   4. Finding it very difficult on present income
4. Education:
   1. None
   2. Elementary education
   3. High School Education
   4. Higher Education
5. What is the number of people living in your household?
6. 1
7. 2
8. 3
9. 4
10. 5
11. 6
12. 7
13. 8
14. 9
15. 10
16. >10
17. What is the number of people living in your household that have a source of income (bring money home):
18. 0
19. 1
20. 2
21. 3
22. 4
23. 5
24. 6
25. 7
26. 8
27. 9
28. 10
29. > 10
30. Which of these descriptions applies to what you have been doing just before finding out you are pregnant??
    1. In paid work (or away temporarily) (employee, self-employed, working for your family business)
    2. In education, (not paid for by employer) even if on vacation
    3. Unemployed and actively looking for a job
    4. Unemployed, wanting a job but not actively looking for a job
    5. Permanently sick or disabled
    6. In community or military service
    7. Doing housework, looking after children or other persons
31. Which of these descriptions applies to your current employment situation?
    1. In paid work (or away temporarily) (employee, self-employed, working for your family business)
    2. In education, (not paid for by employer) even if on vacation
    3. Unemployed and actively looking for a job
    4. Unemployed, wanting a job but not actively looking for a job
    5. Permanently sick or disabled
    6. In community or military service
    7. Doing housework, looking after children or other persons
32. Relationship status:
    1. Married
    2. In a relationship
    3. Single
    4. Widowed
33. Do you feel supported by your partner during this pregnancy?
    1. Yes
    2. No
34. Do you feel supported by other family members or friends during this pregnancy?
    1. Yes
    2. No
35. Were you born in the country that you are living in now?
    1. Yes
    2. No
36. What year did you first come to live in [country]? <type in or selected from list>

**PREGNANCY RISK ASSESSMENT QUESTIONS:**

1. Please enter your height (cm) (answer from number list)
2. Please enter your current weight (kg) (answer from number list)
3. Please enter your weight before the pregnancy (kg) (answer from number list)
4. Is this your first pregnancy?
   1. Yes
   2. No

If YES move up to Q 23

1. How many vaginal deliveries have you had?
   1. 0
   2. 1
   3. 2
   4. 3
   5. 4
   6. 5
   7. 6
   8. 7
   9. 8
   10. 9
   11. 10
   12. >10
2. How many Caesarean section have you had?
   1. 0
   2. 1
   3. 2
   4. 3
   5. 4
   6. 5
   7. 6
   8. 7
   9. 8
   10. 9
   11. 10
   12. >10
3. How many times have you been pregnant?
   1. 1
   2. 2
   3. 3
   4. 4
   5. 5
   6. 6
   7. 7
   8. 8
   9. 9
4. How many pregnancies have you lost before 22 weeks?
   1. 0
   2. 1
   3. 2
   4. 3
   5. 4
   6. 5
   7. 6
   8. 7
   9. 8
   10. 9
   11. 10
   12. > 10
5. How many pregnancies have you lost > 22 weeks?
   1. 0
   2. 1
   3. 2
   4. 3
   5. 4
   6. 5
   7. 6
   8. 7
   9. 8
   10. 9
   11. 10
   12. >10
6. Do you have any pre-pregnancy chronic conditions? (you can select multiple choices)
   1. Hypertension
   2. Diabetes mellitus type 1
   3. Diabetes mellitus type 2
   4. Hypothyroidism or Hashimoto disease
   5. Hyperthyroidism or Graves-Basedow disease
   6. Other endocrinologic diseases (e.g., Adrenal insufficiency or Cushing syndrome, Conn syndrome or primary hyperaldosteronism, adrenal insufficiency or Addison's disease, pituitary diseases)
   7. Polycystic ovary syndrome
   8. Previous oncology treatment
   9. Current oncology treatment
   10. Cardiovascular diseases
   11. Cardiac failure
   12. Congenital disorder
   13. Chronic anaemia
   14. Kidney failure or other kidneys diseases
   15. Systemic lupus erythematosus, polyarthritis rheumatoid or other rheumatic diseases
   16. Healthy before pregnancy
7. Do you have any pregnancy-related conditions or problems during your current pregnancy? (you can select multiple choices)
   1. Pregnancy hypertension
   2. HELLP syndrome
   3. Preeclampsia
   4. Obstetric cholestasis
   5. Gestational diabetes mellitus
   6. Fetal structural abnormalities
   7. Fetus affected by genetic syndromes (e.g. Down, Turner, Edwards, Patau, and others)
   8. Hyperemesis gravidarum
   9. Threatened preterm birth
   10. Threatened miscarriage
   11. Acute fatty liver syndrome
   12. Anaemia during pregnancy treated with iron supplementation
   13. Polyhydramnios
   14. Oligohydramnios
   15. Fetal growth restriction
   16. I do not have any pregnancy-related conditions
8. Have you been told by your doctor/midwife that your pregnancy in a high-risk pregnancy?
   1. Yes
   2. No
9. Have you been treated in an infertility clinic before this pregnancy?
   1. Yes
   2. No
10. Is this pregnancy a result of fertility treatment?
    1. Yes
    2. No
11. How many children are you carrying?
    1. 1
    2. 2
    3. 3
    4. >3

**MENTAL HEALTH HISTORY:**

1. **Before pregnancy** have you every sought any mental health support?
   1. Yes
   2. No
2. **Before pregnancy** have you had any psychiatric treatment?
   1. Yes, pharmacologic
   2. Yes, psychotherapy
   3. Yes, psychotherapy and pharmacologic
   4. No
3. **During this pregnancy** have you sought any mental health support?
   1. Yes
   2. No
4. **During this pregnancy** have received/are you receiving any psychiatric treatment?
   1. Yes, pharmacologic
   2. Yes, psychotherapy
   3. Yes, psychotherapy and pharmacologic
   4. No

**COVID – 19 SPECIFIC QUESTIONS**

1. Have you been infected with the new coronavirus (known as COVID-19) **before pregnancy**?
   1. Yes
   2. No
2. Have you been infected with COVID-19 **during this pregnancy**?
   1. Yes
   2. No
3. How would you rate your level of fear that you or the people close to you will become infected with COVID-19?
   1. 0 (Not fear at all)
   2. 1
   3. 2
   4. 3
   5. 4
   6. 5
   7. 6
   8. 7
   9. 8
   10. 9
   11. 10 (Extreme fear)
4. How much do you feel restricted due to social distancing recommended/implemented during the COVID-19 pandemic?
   1. 0 (Not restricted at all)
   2. 1
   3. 2
   4. 3
   5. 4
   6. 5
   7. 6
   8. 7
   9. 8
   10. 9
   11. 10 (Very restricted)
5. Which of the following imposed restrictions resulting from the COVID-19 pandemic you feel have burdened you the most?
   1. None
   2. I have to give up on my leisure activities
   3. I have to give up on social meetings
   4. I have to work from home
   5. I cannot work at all
   6. I cannot leave the house at all
6. How burdened do you feel by the current COVID-19 pandemic in regard to your/your family members’ possibility to work and earn money (i.e. has it changed because of the pandemic)?
   1. 0 (Not burdened at all)
   2. 1
   3. 2
   4. 3
   5. 4
   6. 5
   7. 6
   8. 7
   9. 8
   10. 9
   11. 10 (Very burdened)
7. How burdened do you feel by the current COVID-19 pandemic in regard to your favourite leisure activities (i.e. has it changed because of the pandemic)?
   1. 0 (Not burdened at all)
   2. 1
   3. 2
   4. 3
   5. 4
   6. 5
   7. 6
   8. 7
   9. 8
   10. 9
   11. 10 (Very burdened)
8. How burdened do you feel by the current COVID19 pandemic in regard to the provision of childcare - closed schools, kindergartens, nurseries, etc. (i.e. has it changed because of the pandemic)?
   1. 0 (Not burdened at all)
   2. 1
   3. 2
   4. 3
   5. 4
   6. 5
   7. 6
   8. 7
   9. 8
   10. 9
   11. 10 (Very burdened)
9. How burdened do you feel by the current COVID19 pandemic in regard to how it has affected your household’s financial situation?
   1. 0 (Not burdened at all)
   2. 1
   3. 2
   4. 3
   5. 4
   6. 5
   7. 6
   8. 7
   9. 8
   10. 9
   11. 10 (Very burdened)
10. How much are you concerned about your unborn child’s safety due to the COVID19 pandemic?
    1. 0 (Not fear at all)
    2. 1
    3. 2
    4. 3
    5. 4
    6. 5
    7. 6
    8. 7
    9. 8
    10. 9
    11. 10 (Extreme fear)
11. How much are you concerned about your family members getting sick and have the adverse effects of the COVID-19?
    1. 0 (Not fear at all)
    2. 1
    3. 2
    4. 3
    5. 4
    6. 5
    7. 6
    8. 7
    9. 8
    10. 9
    11. 10 (Extreme fear)
12. How much are you concerned about you getting sick and having the adverse effects of the COVID-19?
    1. 0 (Not fear at all)
    2. 1
    3. 2
    4. 3
    5. 4
    6. 5
    7. 6
    8. 7
    9. 8
    10. 9
    11. 10 (Extreme fear)
13. How much do you fear that the COVID-19 pandemic will result in restrictions related to your childbirth (presence of accompanying person/s at hospital etc.)
    1. 0 (Not fear at all)
    2. 1
    3. 2
    4. 3
    5. 4
    6. 5
    7. 6
    8. 7
    9. 8
    10. 9
    11. 10 (Extreme fear)
14. How much do you feel burdened by restrictions imposed on labour and delivery as a result of the COVID-19 pandemic (presence of accompanying person/s at hospital etc.)?
    1. 0 (Not burdened at all)
    2. 1
    3. 2
    4. 3
    5. 4
    6. 5
    7. 6
    8. 7
    9. 8
    10. 9
    11. 10 (Very burdened)
15. How much do you fear that your baby will become ill during/after delivery and will have adverse outcomes due to the COVID-19?
    1. 0 (Not fear at all)
    2. 1
    3. 2
    4. 3
    5. 4
    6. 5
    7. 6
    8. 7
    9. 8
    10. 9
    11. 10 (Extreme fear)
16. How much do you fear that your partner will not be able to be present during the delivery?
    1. 0 (Not fear at all)
    2. 1
    3. 2
    4. 3
    5. 4
    6. 5
    7. 6
    8. 7
    9. 8
    10. 9
    11. 10 (Extreme fear)
17. Are you considering home birth because your partner cannot be present with you at the hospital during labour? Choose the answer that best describes your attitude towards home birth.
    1. This is not my case, my country’s policies allow accompanied labour
    2. I have planned home birth regardless of the COVID-19 pandemic
    3. I have previously delivered at home and am planning on doing it this pregnancy as well
    4. My country’s policies have restricted accompanied birth but I have not discussed the possibility of home birth yet
    5. I have never considered and will not consider home birth
    6. I cannot consider home birth because I have a high-risk pregnancy
    7. I have considered home birth but my husband/partner/family is not allowing it for fear of my well being
    8. My husband/partner/family wants us to deliver at home but I am afraid of it
18. How do you view your country’s policies related to the COVID-19 pandemic? Which statement best describes your view/feeling/fear?
    1. They are sufficient and I feel they are aimed at protecting me and my unborn child
    2. The restrictions are not sufficient enough, I fear for myself and my unborn child
    3. I feel the restrictions such as labour without an accompanying person are harmful to me and my child
    4. I fear that I will have to have a Caesarean section if I have suspected/confirmed COVID 19 infection
    5. I fear that if I have suspected/confirmed COVID19 infection I will be separated from my child
    6. I fear that if I have suspected/confirmed COVID19 infection I will not be allowed to breastfeed
19. Which is your number one source of information about COVID-19 pandemic and the new coronavirus?
    1. Social media
    2. Internet published statistics
    3. Medical research papers
    4. Medical provider, general practitioner or midwife that I attend
    5. Family or friends
    6. Newspaper
    7. TV

**PhQ-9**

Over the last two weeks, how often have you been bothered by the following problem?

1. Little interest or pleasure in doing things?
   1. 0 (Not at all)
   2. 1 (Several days)
   3. 2 (More than half the days)
   4. 3 (Nearly every day)
2. Feeling down, depressed, or hopeless?
   1. 0 (Not at all)
   2. 1 (Several days)
   3. 2 (More than half the days)
   4. 3 (Nearly every day)
3. Trouble falling or staying asleep, or sleeping too much?
   1. 0 (Not at all)
   2. 1 (Several days)
   3. 2 (More than half the days)
   4. 3 (Nearly every day)
4. Feeling tired or having little energy?
   1. 0 (Not at all)
   2. 1 (Several days)
   3. 2 (More than half the days)
   4. 3 (Nearly every day)
5. Poor appetite or overeating?
   1. 0 (Not at all)
   2. 1 (Several days)
   3. 2 (More than half the days)
   4. 3 (Nearly every day)
6. Feeling bad about yourself — or that you are a failure or have let yourself or your family down?
   1. 0 (Not at all)
   2. 1 (Several days)
   3. 2 (More than half the days)
   4. 3 (Nearly every day)
7. Trouble concentrating on things, such as reading the newspaper or watching television?
   1. 0 (Not at all)
   2. 1 (Several days)
   3. 2 (More than half the days)
   4. 3 (Nearly every day)
8. Moving or speaking so slowly that other people could have noticed? Or so fidgety or restless that you have been moving a lot more than usual?
   1. 0 (Not at all)
   2. 1 (Several days)
   3. 2 (More than half the days)
   4. 3 (Nearly every day)
9. Thoughts that you would be better off dead, or thoughts of hurting yourself in some way?
   1. 0 (Not at all)
   2. 1 (Several days)
   3. 2 (More than half the days)
   4. 3 (Nearly every day)

**GAD-7**

Over the last two weeks, how often have you been bothered by the following problem?

1. Feeling nervous, anxious or on edge
   1. 0 (Not at all)
   2. 1 (Several days)
   3. 2 (More than half the days)
   4. 3 (Nearly every day)
2. Not being able to stop or control worrying
   1. 0 (Not at all)
   2. 1 (Several days)
   3. 2 (More than half the days)
   4. 3 (Nearly every day)
3. Worrying too much about different things
   1. 0 (Not at all)
   2. 1 (Several days)
   3. 2 (More than half the days)
   4. 3 (Nearly every day)
4. Trouble relaxing
   1. 0 (Not at all)
   2. 1 (Several days)
   3. 2 (More than half the days)
   4. 3 (Nearly every day)
5. Being so restless that it is hard to sit still
   1. 0 (Not at all)
   2. 1 (Several days)
   3. 2 (More than half the days)
   4. 3 (Nearly every day)
6. Becoming easily annoyed or irritated
   1. 0 (Not at all)
   2. 1 (Several days)
   3. 2 (More than half the days)
   4. 3 (Nearly every day)
7. Feeling afraid as if something awful might happen
   1. 0 (Not at all)
   2. 1 (Several days)
   3. 2 (More than half the days)
   4. 3 (Nearly every day)
8. If You checked off any problems, how difficult have these problems made it for You to do Your work, take care of things at home, or get along with other people?
   1. 0 (Not difficult at all)
   2. 1 (Somewhat difficult)
   3. 2 (Very difficult)
   4. 3 (Extremely difficult)
9. When did the symptoms begin? <date from calendar>

**End of survey:**

This is the end of the survey. Thank you for your time. It is very much appreciated by our research team. By clicking the SUBMIT button you are providing consent for the information/data you have provided to be saved and collected in our database for research purposes to be used by our research team.

Once you click the SUBMIT button you will not be able to withdraw your answers. Remember that the survey is anonymous, and the researchers will not be able to identify which data is yours. The data will be stored for 10 years in accordance with the institution's ethics policies.

**Optional Survey Contact Information Form***

Name:

Email:

I would like to be contacted by the research team to be invited to participate in other research projects related to maternal mental health and the COVID-19 pandemic.

1. Yes
2. No

I would like to receive updates about these projects, including results from this survey via the provided email.

1. Yes
2. No

*Note: This information is collected as a separate project in REDCap and is not linked with data from the study’s primary survey

Thank you for your time!
